# Development of a Novel Optical Biosensor for Detection of Organophoshorus Pesticides Based on Methyl Parathion Hydrolase Immobilized by Metal-Chelate Affinity

**DOI:** 10.3390/s120708477

**Published:** 2012-06-25

**Authors:** Wensheng Lan, Guoping Chen, Feng Cui, Feng Tan, Ran Liu, Maolidan Yushupujiang

**Affiliations:** 1 State Key Laboratory of ASIC & System and School of Microelectronics, Fudan University, 220 Handan Road, Shanghai 200433, China; E-Mails: rliu@fudan.edu.cn (R.L.); 06300720461@fudan.edu.cn (M.Y.); 2 Shenzhen R&D Key Laboratory of Exotic Pest Detection Technology, Animal & Plant Inspection and Quarantine Technical Center, Shenzhen Entry-Exit Inspection and Quarantine Burea, 1011 Fuqiang Road, Shenzhen 518045, China; E-Mail: lanwshao@yahoo.com.cn; 3 State Key Laboratory of Integrated Management of Pest Insects and Rodents, Institute of Zoology, Chinese Academy of Science, Beijing 100101, China; E-Mails: cuif@ioz.ac.cn (F.C.); tanfeng0402@163.com (F.T.)

**Keywords:** optical biosensor, methyl parathion hydrolase, organophosphorus compound, metal-chelate affinity

## Abstract

We have developed a novel optical biosensor device using recombinant methyl parathion hydrolase (MPH) enzyme immobilized on agarose by metal-chelate affinity to detect organophosphorus (OP) compounds with a nitrophenyl group. The biosensor principle is based on the optical measurement of the product of OP catalysis by MPH (*p*-nitrophenol). Briefly, MPH containing six sequential histidines (6× His tag) at its *N*-terminal was bound to nitrilotriacetic acid (NTA) agarose with Ni ions, resulting in the flexible immobilization of the bio-reaction platform. The optical biosensing system consisted of two light-emitting diodes (LEDs) and one photodiode. The LED that emitted light at the wavelength of the maximum absorption for *p*-nitrophenol served as the signal light, while the other LED that showed no absorbance served as the reference light. The optical sensing system detected absorbance that was linearly correlated to methyl parathion (MP) concentration and the detection limit was estimated to be 4 μM. Sensor hysteresis was investigated and the results showed that at lower concentration range of MP the difference got from the opposite process curves was very small. With its easy immobilization of enzymes and simple design in structure, the system has the potential for development into a practical portable detector for field applications.

## 1. Introduction

Organophosphorus (OP) compounds, such as methyl parathion (MP), are among the most widely used pesticides in the agricultural industry, home hygiene, garden management and veterinary practice as an alternative to organochlorine chemicals [1–3]. OP compounds strongly inhibit acetylcholinesterase (AChE) activity in animals and pests. AChE degrades the neurotransmitter acetylcholine into choline and acetate, and its inactivation results in acute or chronic dysfunction of the central nervous system [4]. With their extensive application over the past two decades, OP compounds are increasingly causing environmental problems such as contamination of water, vegetables, food and soil, thus becoming a great threat to human health and ecosystem balance. This indicates the importance of developing innovative methods for the effective detection of OP compounds.

Many analytical methods for OP compound detection include gas chromatography, high-performance liquid chromatography and capillary electrophoresis [5]. These methods are used widely as criteria for inspection and quarantine because of their high sensitivity and specificity. However, these methods heavily depend on expensive instruments, time-consuming pretreatments, high-cost reagents and highly qualified technicians, and are not applicable in field monitoring. Biosensors based on specific biomolecular recognition, such as enzyme-substrate, antigen-antibody and DNA/DNA complexes, are suitable candidates for field testing because of their high specificity and sensitivity. Among the different biosensors, enzymatic analysis has been extensively investigated for OP compound monitoring. Two types of enzyme-based analytical methods have been widely studied: indirect quantitative detection of OP compounds using AChE and direct monitoring of OP compounds using organophosphorus hydrolase (OPH). Indirect quantitation involves the measurement of the inhibition rate of AChE activity after exposure to OP compounds [6–12]. Special substrates (e.g., acetylcholine or acetylthiocholine) are used to investigate the inhibition rate, which corresponds to the concentration of OP compounds. Enzyme inhibition in these systems is irreversible in most cases and could not be used for rapid and repetitive measurements in field analysis. By contrast, direct monitoring is concerned with the hydrolysis of OP compounds catalyzed by OPH without the addition of other substrates [13–21]. These enzyme activities are not affected by substrates and could thus be used repeatedly. This method is more applicable and convenient in field analysis and several studies have reviewed the progress of biosensor development for OP compound analysis [22–24]. The present study focused on optical biosensors based on methyl parathion hydrolase (MPH).

The immobilization of biorecognition elements plays a key role in biosensor construction [25]. Several methods, such as adsorption, entrapment, cross-linking and covalent binding, enable the immobilization of biomolecules on the solid surface of a transducer and are widely used in biosensor development. Adsorption is mild and easy to perform, and maintains good enzymatic activity, but enzyme leakage often occurs due to its weak immobilization. Entrapment and cross-linking can immobilize biomolecules tightly; however, randomly deposited enzymes result in partial hindrance of biological activity and possible diffusion barriers for substrates into the biological activity center. Covalent binding enables ordered enzyme immobilization and provides a stable and highly active modified sensing membrane. However, this method utilizes harsh chemical reactions leading to a significant loss of enzyme activity. None of the abovementioned immobilization strategies can produce a stable and regenerable bioactive sensing layer.

In the present study we have developed a novel MPH-based optical biosensor based on the metal-chelate nitrilotriacetic acid (NTA) affinity method. The detection principle is based on the absorbance measurement of the product (*p*-nitrophenol) of MP catalysis by MPH. Recombinant MPH enzyme tagged with six histidines at the *N*-terminal was generated via the molecular cloning method [26]. The enzyme was then anchored on agarose, which was used as solid support, by chelation of the six histidines with Ni^2+^. The enzymatic product was filtrated into the optical cell using a home-made filtration system and was then detected by the optical sensing system. The proposed biosensing system utilized a short and mild immobilization procedure that did not affect the enzyme activity. More importantly, getting a fresh biosensing layer was very convenient through the removal of the degenerated enzyme using a high concentration imidazole solution and the addition of the fresh enzyme. Overall, the proposed system represents a low-cost and portable optical biosensor for field monitoring of OP compounds.

Some studies have reported previously developed biosensors using AChE for the detection of OP compounds with metal-chelate NTA affinity [6,12]. These methods are indirect and are based on electrochemical or piezoelectric analysis. To our knowledge, few studies have focused on metal-chelate biosensors based on MPH until now, which is used as direct analysis.

## 2. Experimental

### 2.1. Materials

Potassium hydrogen phthalate (PHP, pH 4.0), phosphate buffer solution (PBS, pH 6.86) and sodium tetraborate (ST, pH 9.18) were obtained from Shanghai Hongbei Reagent Co., Ltd. (Shanghai, China) as pH buffers. Methyl parathion from Sigma (St. Louis, MO, USA) was used to prepare a stock solution (0.38 mM) by mixing with ethanol and water (1:9) and was stored at 4 °C. Methyl parathion solutions at different pH were obtained by dilution of the stock solution in the corresponding pH buffers. Two LEDs with wavelength of 400 and 610 nm were purchased from a domestic electric supermarket. Ni-NTA agarose stored in ethanol was obtained from Qiagen (Valencia, CA, USA) with a loading capacity of more than 20 mg of 6× His-tagged protein (50 kD).

The gene encoding methylparathion hydrolase (*mpd*) was cloned from *Stenotrophomonas* sp. isolated from soil near the Tian Jing Insecticide Factory. The gene was amplified, purified and then cloned to the vector pET30a (pET-mpd, with six sequential histidines at the *N*-terminal of *mpd*) with a highly efficient expression. The recombinant vector was then transferred to *Escherichia coli* BL21 (DE3), which efficiently expressed MPH with a six histidine *N*-tag. The recombinant bacterial culture was induced by isopropyl β-D-1-thiogalactopyranoside (IPTG) and further incubated under 30 °C for 6 h. After harvesting by centrifugation, the bacterial pellet was diluted using PBS. The bacterial solution was sonicated under ice-bath conditions and centrifuged. The supernate containing the raw enzyme was further purified by successive precipitation of the cell extract. The specific activity of the purified enzyme was 40 units/mL. The refined enzyme was mixed with glycerol (30%, v/v) and stored at −20 °C for further biosensing detection. The properties of purified MPH have been extensively reported in the literature [26–29].

### 2.2. Principle of Detection

**Scheme 1.**
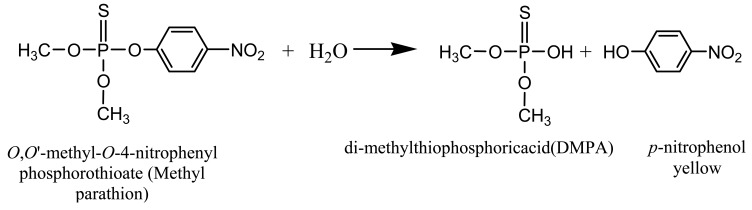
MPH enzymatic reaction of OP compounds.

The biosensor is based on the principle of absorbance measurement of the yellow enzymatic product, *p*-nitrophenol, resulting from the catalysis of colorless MP by MPH [8,20] (Scheme 1).

**Figure 1.**
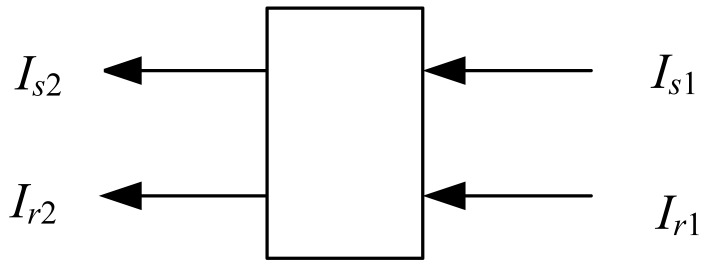
Schematic diagram of two beams passing through a cuvette with width *l*.

Two beams from the LEDs pass through liquid solutions with optical path (Figure 1). The signal light (*I_s_*_1_) corresponds to the maximum absorption wavelength of the enzymatic product at 400 nm, whereas the reference light (*I_r_*_1_) is seldom absorbed by the enzymatic product.

The transmitted light (*I_s_*_2_ and *I_r_*_2_) are collected by a photodiode and converted into an electronic signal. The transmission is expressed in terms of the absorbance of the liquid solution as follows:

(1) $$A_{s}=\mathrm{lg}(I_{s1}/I_{s2})=c\varepsilon_{s}l$$

(2) $$A_{r}=\mathrm{lg}(I_{r1}/I_{r2})=c\varepsilon_{r}l$$

where *A_s_* and *A_r_* are the absorbance of the signal and reference light, respectively; *c* is the concentration of an absorbing substance; and *ε_r_* and *ε_s_* are the molecular absorptive coefficients of the reference and signal light, respectively. According to Equations (1) and (2), the logarithm of the ratio of intensity of the transmitted reference light to that of the transmitted signal light is linearly correlated to the concentration of the absorbing substance, as follows:

(3) $$\mathrm{lg}(I_{r2}/I_{s2})=\mathrm{lg}(I_{r1}/I_{s1})+c(\varepsilon_{s}-\varepsilon_{r})l$$

### 2.3. Optical Biosensing Design

**Figure 2.**
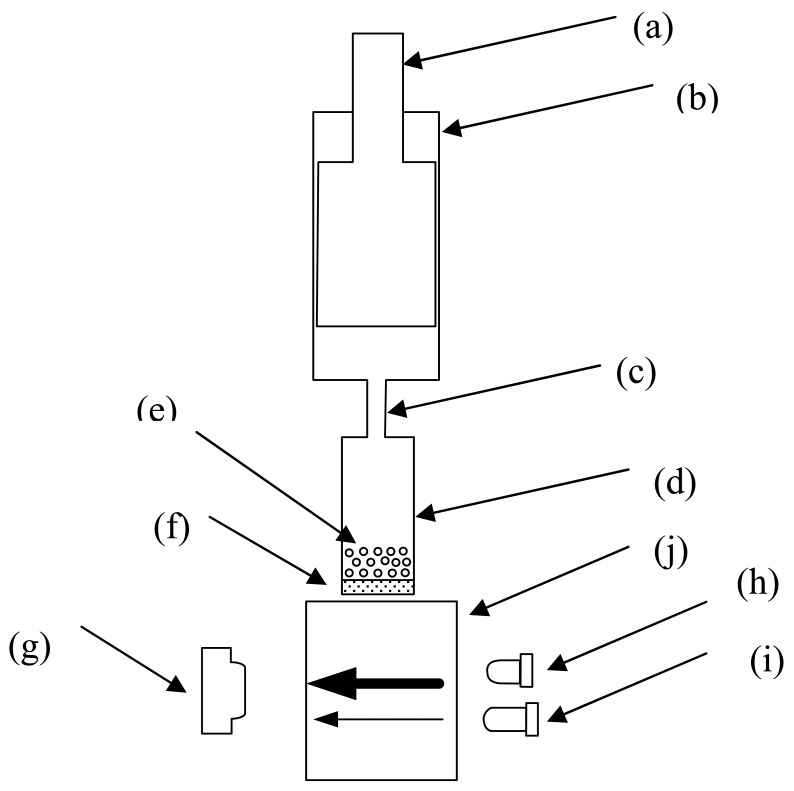
Schematic diagram of the proposed optical biosensor: (**a**) plunger; (**b**) upper chamber; (**c**) middle chamber; (**d**) bottom chamber; (**e**) Ni-NTA; (**f**) filtrating membrane; (**g**) PIN-FET; (**h**) blue LED with wavelength of 400 nm; (**i**) red LED with wavelength of 610 nm; and (**j**) optical cell.

The optical biosensing system is composed of a filtering component and an optical sensing component (Figure 2). The plastic filtering component was designed to separate the liquid from the agarose into the optical cell for the detection of OP compounds. It contains three cylindrical chambers of varied sizes. The upper chamber is a 2 mL tube with an 8.6 mm inner diameter. A plunger fits into the upper chamber tightly and can be pushed and pulled along the inside wall of the chamber. The bottom chamber is approximately 45 μL with a 7.4 mm inner diameter with a polyester filtrating membrane with 0.45 μm pores at the bottom. The enzymatic reaction occurred in the bottom chamber. After filling the bottom chamber with Ni-NTA agarose, the plunger of the upper chamber was pushed and pulled slowly for several times and the liquid was filtrated through the filtrating membrane. The middle chamber between the upper chamber and the bottom chamber has a 2.5 mm inner diameter. It was designed to be small enough to avoid the reflux of agarose into the upper chamber from the bottom chamber when the plunger was pulled inside the upper chamber. The filtrated liquid was collected into the optical cell with a 1 cm optical path and was detected by the optical sensing component for absorbance measurement.

The optical sensing component contained two LEDs as optical sources. One LED emitted blue light at a wavelength of 400 nm, which corresponded to the maximum absorption of *p*-nitrophenol, and served as the signal source. The other LED emitted red light at a wavelength of 610 nm, at which no absorption occurred for *p*-nitrophenol, and was used as the reference source. The two LEDs emitted light alternately and their beams were transmitted through the optical cell. The signal source has 25 nm full-width at half-maximum (FWHM) with about 100 mW power consumption whereas the reference source has about 40 nm FWHM and 20 mW power consumption. A PIN-FET photodiode (L20, Suo Yang Scientific Company, Beijing, China) in the opposite side of the two LEDs collected the light emission and converted it into electronic signals for the subsequent absorbance analysis.

### 2.4. Immobilization of MPH and the Analytical Process

**Scheme 2.**
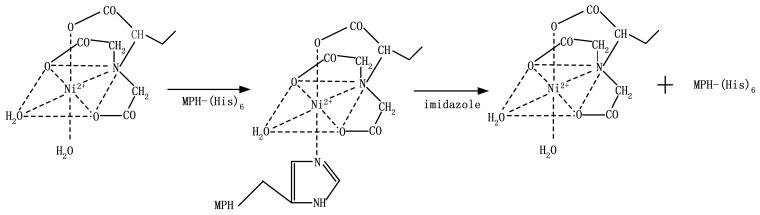
MPH immobilization and separation scheme.

Metal-chelate affinity-based immobilization was used in the proposed system. The immobilization method takes advantage of the ability of Ni^2+^ to bind histidine residues at the tail of the protein [6]. Ni^2+^ has six chelation sites: four for the NTA molecule covalently bonded to agarose, one for H_2_O, and one for the histidine at the tail of the MPH enzyme molecule. The immobilization scheme is presented in Scheme 2 [30].

To immobilize MPH, the Ni-NTA agarose stored in ethanol was filled into the bottom chamber of the filtering component after the plunger was pulled out. Then, the plunger was inserted into the upper chamber of the filtering component and pulled down. The filtering component is hermetic except at the exit of the bottom chamber, where a filter membrane is positioned. Ethanol was squeezed out during extrusion of the plunger. After the plunger was pulled and pushed for several times, the liquid was filtered from the Ni-NTA agarose. Then, ultra-pure H_2_O was added into the filtering component and Ni-NTA agarose was thoroughly washed thrice. The enzyme solution was poured in and incubated with Ni-NTA agarose for several minutes to immobilize the enzyme on the resin. The remaining enzyme solution was then pumped out. Finally, MP solutions of different concentrations were added to the bottom chamber and were hydrolyzed by the enzymes on the Ni-NTA agarose. After the plunger was carefully pulled and pushed several times, the aqueous solution was filtered from the filtering component and collected into the optical cell. Optical analysis was performed by the optical sensing component for absorbance measurement.

## 3. Results and Discussion

### 3.1. Optical Sensing System

**Figure 3.**
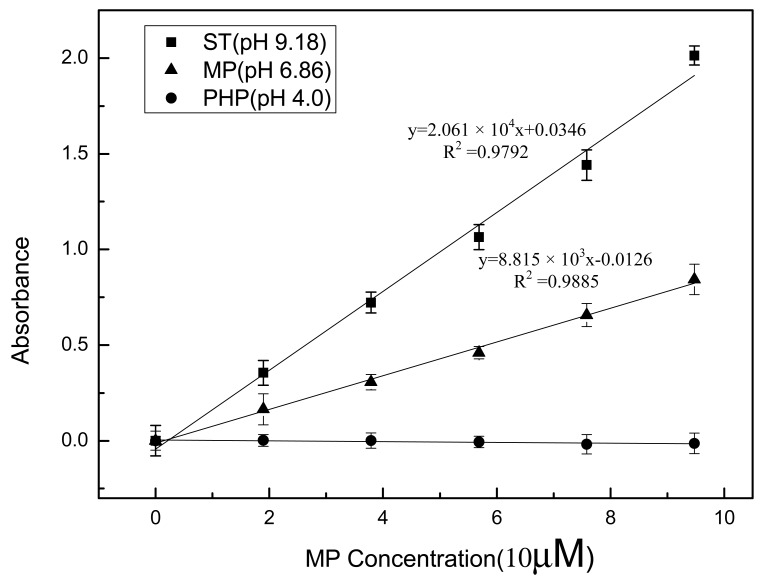
Enzymatic activity of MPH in aqueous solutions against MP concentration in different pH buffers. Three detection times are employed for generating the error bars and the same for the following Figures 4 and 5.

An optical arrangement with double beams can compensate for the effect of temperature fluctuation, electric source instability and external interference on absorbance measurement and is often used in portable optic sensor designs [31,32]. Figure 3 shows the response of the optical sensing system to enzymes mixed with MP in different pH buffer solutions. PHP (pH 4.0), PBS (pH 6.86) and ST (pH 9.18) were used as representative pH buffer solutions. Enzymes were dissolved in buffers mixed with the enzymatic substrate, and were then analyzed using the optical sensing component.

As shown in Figure 3, the absorbance/concentration curves under basic and neutral conditions exhibit a significant linear relationship up to concentration of 1 × 10^−4^ M with a correlation of about 0.99. Furthermore, the absorbance increases more sharply at pH 9.18 than that at pH 6.86 with concentration enhancement. The sensitivity, corresponding to the slope of the regression line [33,34], at pH 9.18 is more than twice than that at pH 6.86. This demonstrates that a basic environment is more favorable for MPH catalysis than a neutral environment. MPH showed almost no catalytic activity and no absorbance was detected at pH 4.0, indicating that highly acid conditions cannot be used for field applications. In fact, reports on the effect of pH on MPH activity are not consistent with each other. Rishpon and co-workers demonstrated that OPH has a broad optimum pH, ranging from pH 8 to pH 10.5 [19]. Another report stated that over 90% of maximum enzyme activity was retained between pH 7 and 9 and approximately 85% of the maximum activity can be maintained at both pH 6.86 and 9.18 [26], which is obviously different from the results in this manuscript. However Yang *et al.* [35] reported that the optimal pH of MPH is 11.0. This discrepancy may be attributed to the different bioengineering methods used in enzyme preparation and the underlying reasons need further investigation.

In Equations (1–3), all lights are considered as special monolights and the absorbance corresponds to a definite wavelength. However, all LEDs used in the current study emitted wide wavelengths and no optical filters were used. The emission spectra of the reference and signal LEDs overlap with the absorbance peak of the enzymatic products. Thus, Equation (3) should be modified as follows [31]:

(4) $$\mathrm{lg}[\left(\int I_{r2}(\lambda )d\lambda \right)/\left(\int I_{s2}(\lambda )d\lambda \right)]=\mathrm{lg}[\left(\int I_{r1}(\lambda )d\lambda \right)/\left(\int I_{s1}(\lambda )d\lambda \right)]+c\varepsilon^{\prime }l$$

where *ε*′ is the equivalent molecular absorptive coefficient corresponding to the ratio of the integral of the reference LED to that of the signal LED. Based on Figure 3, we concluded that an optical system using simple optical elements is applicable for absorbance detection. The results obtained from the procedure demonstrate a good linear relationship between the absorbance and the concentration of the enzymatic product.

### 3.2. Optical Biosensor Response

**Figure 4.**
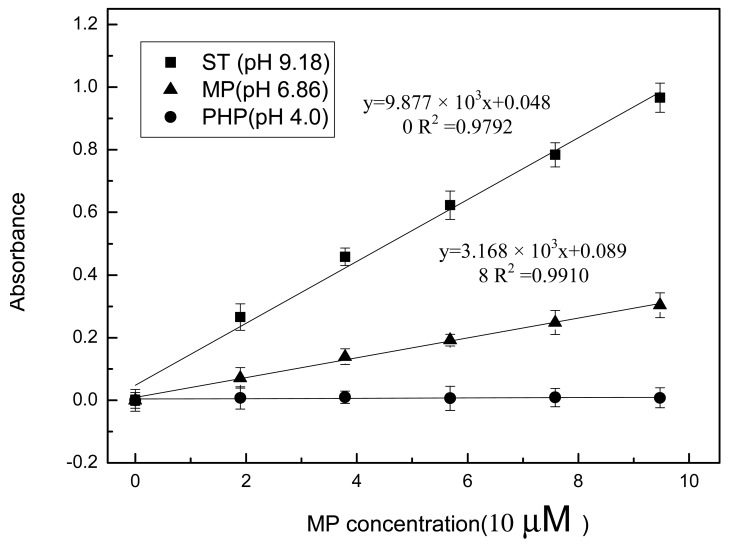
Optical biosensor response at different pH buffers. The enzymes were immobilized on Ni-NTA agarose and the enzymatic products were filtrated into the optical cell using a home-made filtrating system.

**Figure 5.**
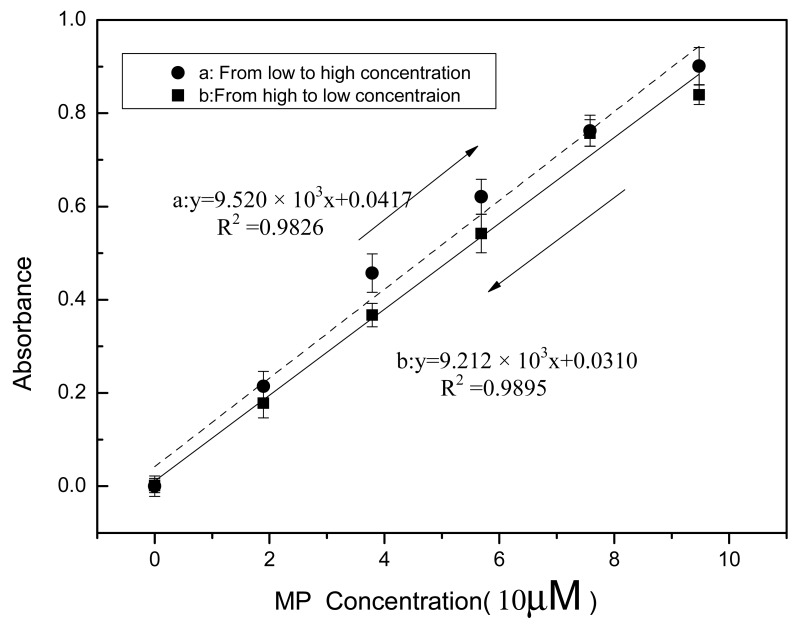
Sensor hysteresis of opposite detection processes.

In the current study, the metal-chelate affinity method was used to immobilize enzyme molecules. The chelator NTA was covalently bound to agarose, which is insoluble in aqueous solutions. Thus, it is impossible to directly detect absorbance through the suspension mixture because of the light scattering from the agarose. A home-made filtering component was used to separate the aqueous enzymatic products from agarose into the optical cell for analysis (Figure 2). After MPH was immobilized on the Ni-NTA agarose, the enzyme/MP mixture was incubated for approximately 15 min to allow full enzymatic reaction in the bottom chamber of the filtering component. Then, the reaction mixture was filtered into the optical cell after the plunger was pulled and pushed. The absorbance of the mixture was measured using the optical sensing component. At the same time, the response of the immobilized enzyme to the MP in different pH buffer solutions was investigated (Figure 4). Similar to the enzymes that were maintained in aqueous form, the calibration curves obtained from the immobilized enzymes showed a good linear relationship between absorbance and MP concentration up to 1.0 × 10^−4^ M at pH 6.86 and 9.18. No absorbance was detected in the pH 4.00 buffer. Moreover, the biosensor exhibited a more sensitive response in basic solution than in neutral environment. However, the slopes of the two calibration curves with enzymes in immobilized form were all less than those in aqueous solution (Figure 5). The difference in form and amount of enzymes in both of these assays may have a contribution to this difference. In aqueous solution, MPH existed in a dissolved state and the high concentration of enzyme molecules were thoroughly mixed with the substrate. But enzyme volume greatly decreased after immobilization on the agarose support and the enzymes had limited contact with the substrate, resulting in decreased enzymatic products. It was also pointed out that the sensitivity of the sensing system begins to decline gradually (data not shown) in both Figures 3 and 4 as pH increases over 9.18.

The limit of detection (LOD) for OP compound monitoring in the environment is one of the most pressing issues in field application and the most researchers are interested in getting the lowest LOD for OP compounds in the environment. Table 1 lists the characteristics of some representative enzyme-based biosensors for OP compound monitoring. As tor ST at pH 9.18, the LOD of our biosensor was estimated to be as low as 4 μM. This LOD is thrice that of the standard deviation of the absorbance measurement for the same buffer without MP. It can be found that electrochemical, piezoelectric and chemiluminescent detection methods can yield lower LODs than these based on optical absorption, pH and fiber optics, which are comparable to the results in this study. However, the method used in this research has special advantages, such as simple immobilization in biomolecular materials, as well as easy regeneration of the biosensing membrane.

### 3.3. Sensor Hysteresis

Sensor hysteresis reflects the differences in sensor outputs obtained from the same input during the two opposite processes, wherein one the input increases and the other the input decreases. It is one of the static properties of physical and chemical sensors, and has been widely discussed in the literature [33,34]. The Ni-NTA agarose support with immobilized MPH was fully washed using ST buffers at pH 9.18 after analysis of each sample. MP samples of different concentrations were analyzed. It took about 25 minutes for each sample measurement. In the MP analysis, the sample concentration was initially increased and finally reduced and it is important to investigate whether a large difference between the results of the two opposite processes would be observed. Figure 5 shows the calibration curves of the absorbance determination from the two opposite processes: curve A shows the trend from low to high concentration, whereas curve B illustrates the opposite trend (from high to low concentration). The absorbance obtained from curve A was higher than that from curve B and with increase in the MP concentration the difference between the two curves became apparent. The lower absorbance obtained from curve B may be attributed to the decreased enzymatic activity after repeated use. For MP with concentration of 5 × 10^−6^ M, the difference in the absorbance gotten from the two curves was estimated to be 0.012 and for absorbance with 0.2, the difference in the concentration gotten from the two curves was estimated to be 1.72 × 10^−6^ M, which is less than the LOD mentioned above. Thus it can be inferred that in the lower detectable range of MP concentrations, the low sensor hysteresis exhibits minimal effects on field OP compound monitoring. Anymore, from curve A to curve B, absorbance increased with the concentration increase and then decreased with the concentration decrease. The results show that the residues from a proceeding sample seem to have a minimal effect on the subsequent sample detection and do not exhibit accumulation effects on sample analysis. To our knowledge, this study is the first to report on biosensor hysteresis.

## 4. Conclusions

In the current study, an optical biosensor for MP detection based on MPH immobilization by metal-chelate affinity was developed. Three representative buffers were chosen to study the pH effect on the biosensor response. Enzymes at pH 9.18 proved to be more suitable than at pH 6.86 for MP concentration monitoring while show no activity at highly acid condition. The optical biosensor we developed can detect MP concentrations as high as 1 × 10^−4^ M and the LOD was estimated to be about 4 × 10^−6^ M, which is comparable to those based on fiber optic and pH detection. Investigation on sensor hysteresis revealed that at lower MP concentrations, the small difference between the opposite process curves had a negligible effect on the sample analysis. In comparison to biosensors based on enzyme inhibition, the optical biosensors based on organophosphate hydrolase can prevent the irreversibility of enzyme activity and promoted a strong anti-interference activity. The proposed optical biosensor is currently being developed into a commercial product for further field applications. The reproducibility and stability of the device was checked by measuring five different batches of samples at the same concentration of 3 × 10^−5^ M and the relative standard deviation (R.S.D) for these samples was measured to be about 3.5%. The possible interferences mainly include stray emission, which still can affect the detection although two LEDs are used in the system, and absorbing substances that show absorption at the wavelength of 405 nm. Temperature, enzymatic activity, electronic source stability and air bubble appearing in the curvette can also affect the detection results of the developing biosensor.

## Figures and Tables

**Table 1.** Selection of enzyme-based biosensors for OP compound detection [12].

**Reference**	**Detection method**	**Enzyme**	**Enzyme immobilization**	**Degree of difficulty in of biorecognition element regeneration**	**LOD**	**Linear range**
[21]	Electrochemical detection	OPH	Absorption	difficult	4 × 10^−7^ M methyl parathion	4.6−5 × 10^−6^ M
[12]	Piezoelectric detection	BChE	Metal-chelation	easy	10 × 10^−9^ M DFP	≈100−10 × 10^−9^ M
[36]	Photoluminescence	OPH	Charge absorption with (CdSe)ZnS quantum dots	difficult	≈10^−8^ M paraoxon	≈10^−5^−10^−7^ M
[37]	Fiber optic detection	OPH	Expressed on *Escherichia coli* cells	difficult	3 × 10^−6^ M paraoxon	0−0.6 × 10^−3^ M
[38]	pH	OPH	Cross-linking	difficult	2 × 10^−6^ M paraoxon	0.15−0.7 × 10^−3^ M
Our method	Optical absorption	OPH	Metal-chelation	easy	4 × 10^−6^ M Methyl parathion	0−1× 10^−4^ M

Abbreviations: OPH, organophosphorus hydrolase; BChE, butyrylcholinesteras; DFP, diisopropylfluorophosphate.
